# Apoptotic cell-mediated suppression of streptococcal cell wall-induced arthritis is associated with alteration of macrophage function and local regulatory T-cell increase: a potential cell-based therapy?

**DOI:** 10.1186/ar2750

**Published:** 2009-07-02

**Authors:** Sylvain Perruche, Philippe Saas, Wanjun Chen

**Affiliations:** 1 Mucosal Immunology Unit, Oral Infection and Immunity Branch, National Institute of Dental and Craniofacial Research, National Institutes of Health, Convent Drive, Bethesda, MD 20892, USA; 2 Inserm UMR645, EFS B/FC, Université of Franche-Comté, IFR133, 1 Bd A. Fleming, 25020 Besancon, France

## Abstract

**Introduction:**

Experimental streptococcal cell wall (SCW)-induced arthritis is characterized by two successive phases of the disease. The acute phase occurs early and is associated with an inflammatory process and neutrophil infiltration into the synovium. The second chronic phase is related to effector T-cell activation and the dysregulation of macrophage function. Creation of an immunomodulatory environment has been attributed to apoptotic cells themselves, apoptotic cell uptake by phagocytes as well as a less sensibility of phagocytes capturing apoptotic bodies to activation. Therefore we evaluated the potential of apoptotic cell injection to influence the course of inflammation in SCW-induced arthritis in rats.

**Methods:**

Rat apoptotic thymocytes were injected intraperitoneally (2 × 10^8^) in addition to an arthritogenic dose of systemic SCW in LEW female rats. Control rats received SCW immunization and PBS. Rats were then followed for arthritis occurrence and circulating cytokine detection. At sacrifice, regulatory T cells (Tregs) and macrophages were analyzed.

**Results:**

Apoptotic cell injection profoundly suppressed joint swelling and destruction typically observed during the acute and chronic phases of SCW-induced arthritis. Synovial inflammatory cell infiltration and bone destruction were also markedly suppressed. *Ex vivo *experiments revealed reduced levels of TNF in cultures of macrophages from rats challenged with SCW in the presence of apoptotic thymocytes as well as reduced macrophage response to lipopolysaccharide. Moreover, apoptotic cell injection induced higher Foxp3^+ ^Tregs in the lymphoid organs, especially in the draining lymph nodes.

**Conclusions:**

Our data indicate that apoptotic cells modulate macrophage function and result in Treg generation/increase. This may be involved in inhibition of inflammation and amelioration of arthritis. This highlights and confirms previous studies showing that *in vivo *generation of Tregs using apoptotic cell injection may be a useful tool to prevent and treat inflammatory autoimmune responses.

## Introduction

The most salient feature of apoptosis is the lack of inflammatory responses or tissue damage. Several mechanisms of peripheral tolerance have been described to explain this lack of immune responses against apoptotic cell-derived antigens [1,2]. First, apoptotic cells themselves possess immunomodulatory properties by the release of transforming growth factor beta (TGFβ) stored in their cytoplasm [3]. Then professional phagocytes, such as macrophages and some dendritic cell subsets [1,4], can also favor an immunomodulatory environment by the release of anti-inflammatory cytokines during apoptotic cell uptake. Such immunomodulatory milieu consists mainly of TGFβ and IL-10 [5-7].

Recently, the role of TGFβ in immune tolerance has been highlighted by its direct and indirect effects on autoimmunity and inflammation [6,8]. Moreover, TGFβ is a key factor to convert peripheral naive CD4^+^CD25^- ^T cells into CD4^+^CD25^+^Foxp3^+ ^regulatory T cells (Tregs), *in vitro *[9] as well as *in vivo *[8]. Also, the TGFβ signaling pathway has also been shown to be critical for natural Treg development [10].

The feasibility of cellular therapy based on the immunomodulatory properties of apoptotic cells has already been evaluated in different experimental models to restore or induce immune tolerance. Indeed, apoptotic cell injection favors allogeneic hematopoietic cell engraftment, favors allograft heart survival and decreases acute graft-versus-host disease (for a review see [11]). Moreover, spontaneous type I diabetes occurrence in NOD mice could be delayed by injection of apoptotic beta cells [12]. These beneficial effects have been mainly related to TGFβ and/or Tregs [11-13].

Although such an approach of apoptotic cell infusion has not yet been used directly in patients, the immunomodulatory properties of apoptotic cells may play a role in the tolerogenic effects of blood product transfusions [14] or of extracorporeal photochemotherapy [15,16]. Indeed, the beneficial effects of extracorporeal photochemotherapy in the treatment of severe chronic or acute graft-versus-host disease have been associated with the significant number of the apoptotic cells generated during extracorporeal photochemotherapy [15,16]. While apoptotic cell instillation prevents and treats autoimmunity [8] and inflammation in several experimental models [6,11,13], the suppressive effect of apoptotic cell infusion on experimental arthritis is unknown.

Injection of Group A streptococcal cell wall (SCW) peptidoglycan–polysaccharide complexes induces an acute inflammation of the peripheral joints, followed by a chronic, erosive arthritis in susceptible rats. This corresponds to an animal model for rheumatoid arthritis (RA) [17,18]. The acute phase is clinically evident within 24 hours after injection of SCW and is characterized histologically by neutrophil infiltration into the synovium. The chronic erosive arthritic stage, on the other hand, is induced by T-cell-mediated and macrophage-mediated immune responses, characterized by accumulation of mononuclear cells with release of proinflammatory cytokines and erosive destruction of subchondral and periarticular bone and cartilage [18-20].

Systemic administrations of IL-4, TGFβ or an inhibitor of nitric oxide have been shown to suppress pathogenesis of SCW arthritis [19,20]. Macrophage depletion could also suppress the chronic phase of the SCW-induced arthritis [21]. Oral administration of SCW prior to systemic injection of SCW substantially prevents the joint swelling and destruction typically observed during both acute and chronic phases of the arthritis [18]. The effect of oral tolerance on SCW arthritis was associated with an increase in circulating levels of TGFβ accompanied by a decrease in inflammatory cytokines and inhibition of the arthritic response [18]. Because macrophages have been identified as pathogenic in SCW-induced RA and because TGFβ has a protective role on SCW-induced RA, we proposed to test the efficiency of apoptotic cell infusion to modulate the arthritic response.

## Materials and methods

### Animals, induction and monitoring of arthritis

Arthritis was induced in pathogen-free Lewis female rats (Charles River Laboratories, Wilmington, MA, USA) by intraperitoneal injection of Group A SCW peptidoglycan–polysaccharide complexes (30 μg rhamnose/g body mass; Lee Laboratories, Grayson, GA, USA) [18]. Animals were housed in a specific pathogen-free rodent facility at the National Institute of Dental and Craniofacial Research, National Institutes of Health. All animal studies were performed according to National Institutes of Health guidelines for use and care of live animals and were approved by the Animal Care and Use Committee of National Institute of Dental and Craniofacial Research.

Acute and chronic joint pathology was clinically monitored and the articular index was determined, as previously described [17,19]. Briefly, the degree of joint swelling was monitored using a plethysmometer (UGO Basile, Varese, Italy). Radiographs taken with direct exposure (1:1) on X-Omat TL Kodak film using 60-kV, 345-mA, 60-s exposure by a Faxitron X-ray machine (Faxitron X-ray Corporation, Buffalo Grove, IL, USA) were evaluated for soft tissue swelling, joint space narrowing, bone erosions and deformity. On days 25 and 26 after SCW immunization, joints were harvested and fixed with neutral 10% formalin, extracted, embedded in paraffin and cut into 5 μm sections for H & E staining.

### Preparation of apoptotic cells

Rat thymocytes were gamma-irradiated (1,500 rad) and cultured in complete DMEM medium at 5% carbon dioxide and 37°C for 4 to 6 hours as previously described [22]. This culture allowed apoptotic changes to occur. Cells were 90 to 95% apoptotic as determined by Annexin-V staining and 7-Aminoactinomycin D exclusion before washing with PBS and intraperitoneal injection into the indicated rats at 2 × 10^8 ^cells per animal at the same time as SCW (two different injections). This corresponds to the early apoptotic state, as indicated by 7-Aminoactinomycin D exclusion. Cells were 70 to 80% apoptotic 3 hours after irradiation and were 90 to 95% apoptotic 6 hours after apoptosis induction.

### Flow cytometry

The spleens, inguinal and mesenteric lymph nodes were removed aseptically and single-cell suspensions were prepared. Peripheral T cells were also analyzed after retro-orbital bleeding and red cell lysis with ACK lysing buffer (Biowhittaker, Walkersville, MD, USA). One to 5 × 10^5 ^cells were resuspended in PBS (Biowhittaker) containing 1% BSA (Irvine, Santa Ana, CA, USA). For surface staining, cells were incubated with FITC-conjugated anti-rat CD4 (Caltag, San Francisco, CA, USA) and allophycoyanin-conjugated anti-CD25 mAbs (BD Biosciences, San Jose, CA, USA) on ice for 30 minutes. After two washes with PBS-% BSA, cells were prepared for intracellular phycoerythrin-labeled Foxp3 mAb staining according to the manufacturer's recommendations (eBiosciences, San Diego, CA, USA). Cells were then resuspended in 0.5 ml PBS-1% BSA for analysis by flow cytometry (FACSCalibur^®^; BD Biosciences) using CellQuest Pro^® ^software (BD Biosciences).

### Cell culture and cytokine assays

Peritoneal macrophages were obtained 4 days after disease induction from peritoneum cavity exudates. Briefly, after four washes with cold PBS of the peritoneum cavity of each rat, enriched macrophage suspension was adjusted to 1 × 10^6 ^cell/ml and cultured with or without lipopolysaccharide (LPS) stimulation (50 ng/ml) in complete DMEM medium containing 10% (vol/vol) heat-inactivated FBS, 2 mM glutamine, 15 mM Hepes, 1% nonessential amino acids, 1 mM sodium pyruvate, penicillin (100 μg/ml), streptomycin (50 μg/ml) and 50 μM 2-mercaptoethanol (all from Biowhittaker). Supernatants were then collected at 24 hours and tested for TNF by ELISA (BioLegend, San Diego, CA, USA) following the manufacturer's instructions. Rats were blood punctured in the retro-orbital sinus at days 1, 4, 6 and 11 for total TGFβ quantification in the plasma after a 1/20 dilution by ELISA (Promega, Madison, WI, USA) following the manufacturer's instructions.

### Statistical analysis

Group comparisons of parametric data were made by Student's *t *test. We used the Mann-Whitney rank-sum test for nonparametric data. We assessed score comparisons between groups by one-way analysis of variance, and when significant differences were found we used Dunn's method to identify differences compared with the control group. We performed statistical analyses with SigmaStat 3.11 software (Systat Software, Richmond, CA, USA). We tested data for normality and variance, and considered *P *< 0.05 significant. Statistical analysis was assessed when the number of experimented animals or conditions was sufficient.

## Results

### Injection of apoptotic cells prevents SCW-induced arthritis in susceptible rats

**Figure 1:**
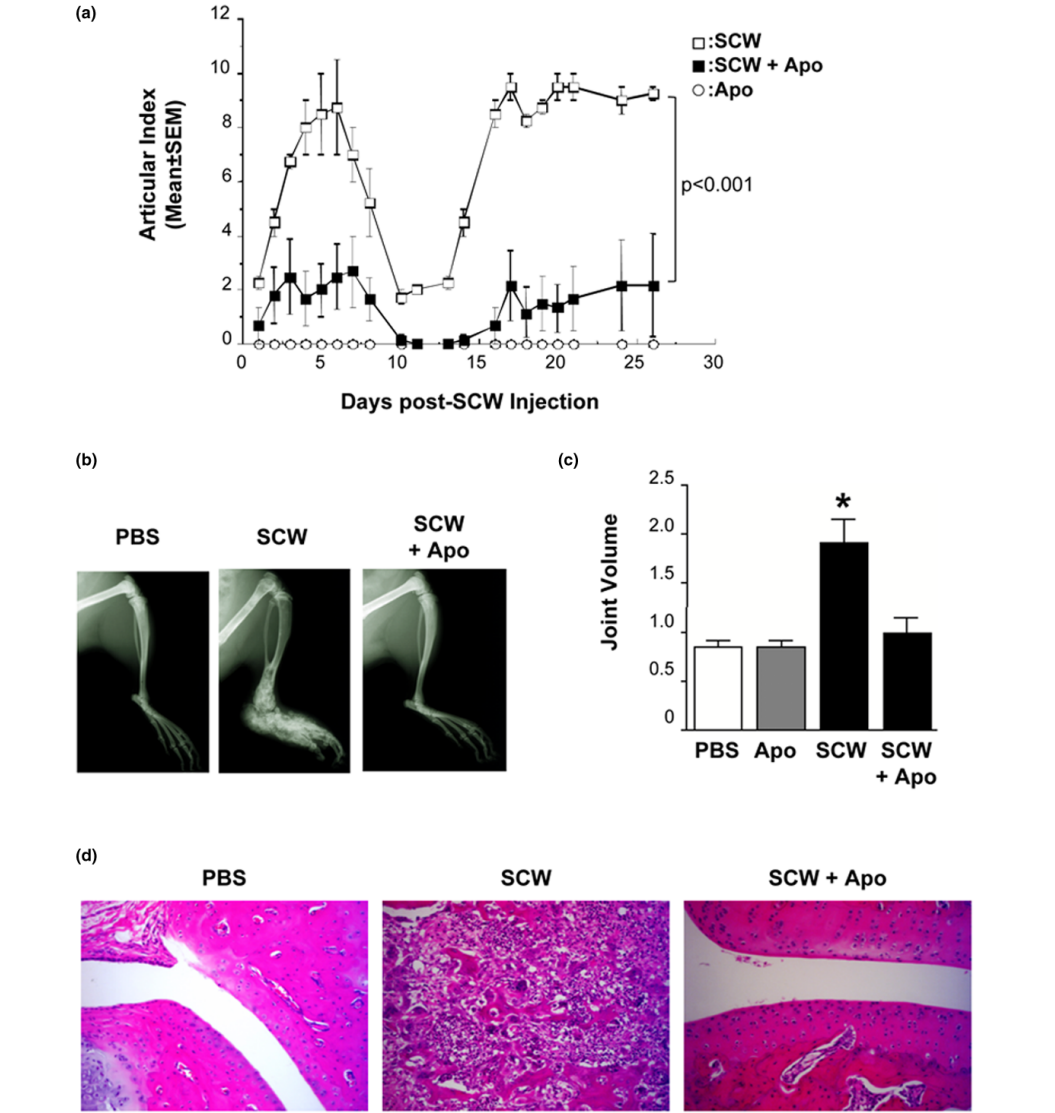
Apoptotic cell injection prevented streptococcal cell wall-induced arthritis. **(a) **Rats were injected with streptococcal cell wall (SCW) in addition to apoptotic cells (Apo, 2 × 10^8 ^cells) or with Apo only and were followed for arthritis occurrence, scored using an articular index for each animal (mean ± standard error of the mean (SEM); n = 3 or 4 rats for each group). *P *< 0.001, SCW vs. SCW + Apo. **(b) **Joint swelling and bone destruction were assessed by X-ray exposure in the different groups (representative animals from each group) as well as **(c) **the joint volume using a plethysmometer, both at day 21 post SCW injection (mean ± SEM; *P < 0.05 vs. PBS, Apo and SCW + Apo). **(d) **H & E analysis of the joints in rats with the indicated treatments at days 25 and 26 post SCW injection. A representative rat from each group is shown (magnification 20×). Each group contained three to six rats. The experiment was repeated three times with similar results.

We first assessed the impact of apoptotic cell injection in a model of inducible arthritis after injection of SCW peptidoglycan–polysaccharide complexes in susceptible Lewis rats. Injection of 3 to 4 mg SCW per rat induced a first acute phase of arthritis for about 6 days after injection, followed by a resolution phase and a chronic phase at around day 15 after immunization (Figure 1a). Injection of apoptotic cells with SCW significantly reduced the severity of the arthritis in both the acute phase and the chronic phase as determined by an articular index (*P *< 0.001 SCW vs. SCW + apoptotic cells; Figure 1a and Table 1). Apoptotic cell injection alone (in the absence of SCW) did not induce any sign of arthritis occurrence (Figure 1a).

**Table 1:** Apoptotic cell injection prevents rats from SCW-induced arthritis development

	Acute phase (day 3)		Remission phase (days 10 and 11)		Chronic phase (days 24 to 30)	
			
	SCW + PBS	SCW + Apo	SCW + PBS	SCW + Apo	SCW + PBS	SCW + Apo
AI score^a^	4.2 ± 0.8	2.0 ± 0.6	1.7 ± 0.7	0.5 ± 0.7	4.9 ± 3.5	1.3 ± 1.4
*n*	12	13	5	6	5	6
*P *value^b^	0.041		0.052		0.030	
Incidence^c^	10/12	7/13	4/5	2/6	5/5	3/6

Pooled results from of three independent experiments. *n*, number of rats. ^a^Articular index score presented as mean ± standard error of the mean. ^b^Compared between streptococcal cell wall (SCW) and SCW + apoptotic cells (Apo); Mann-Whitney rank-sum test, one tail. ^c^Number of rats with disease among the rats of each group; the lower number of animals in the chronic phase is due to sacrifice of animals at the end of the acute phase in some experiments.

The dramatic effect of apoptotic cell injection on the course of SCW-induced arthritis development could also be observed at the level of joint swelling and bone destruction assessed by autoradiography (Figure 1b) or using a plethysmometer (Figure 1c) during the chronic phase of arthritis compare with SCW injection alone. Consistent with the substantial amelioration of the disease score, administration of apoptotic cells also dramatically reduced the synovial inflammatory cell infiltration and bone destruction (Figure 1d).

At the time of arthritis induction by SCW injection, therefore, administration of apoptotic cells significantly decreases the course of arthritis occurrence and the severity of the disease, demonstrating the immunomodulatory properties of apoptotic cells.

### Injection of apoptotic cells decreases the proinflammatory response of macrophages

**Figure 2:**
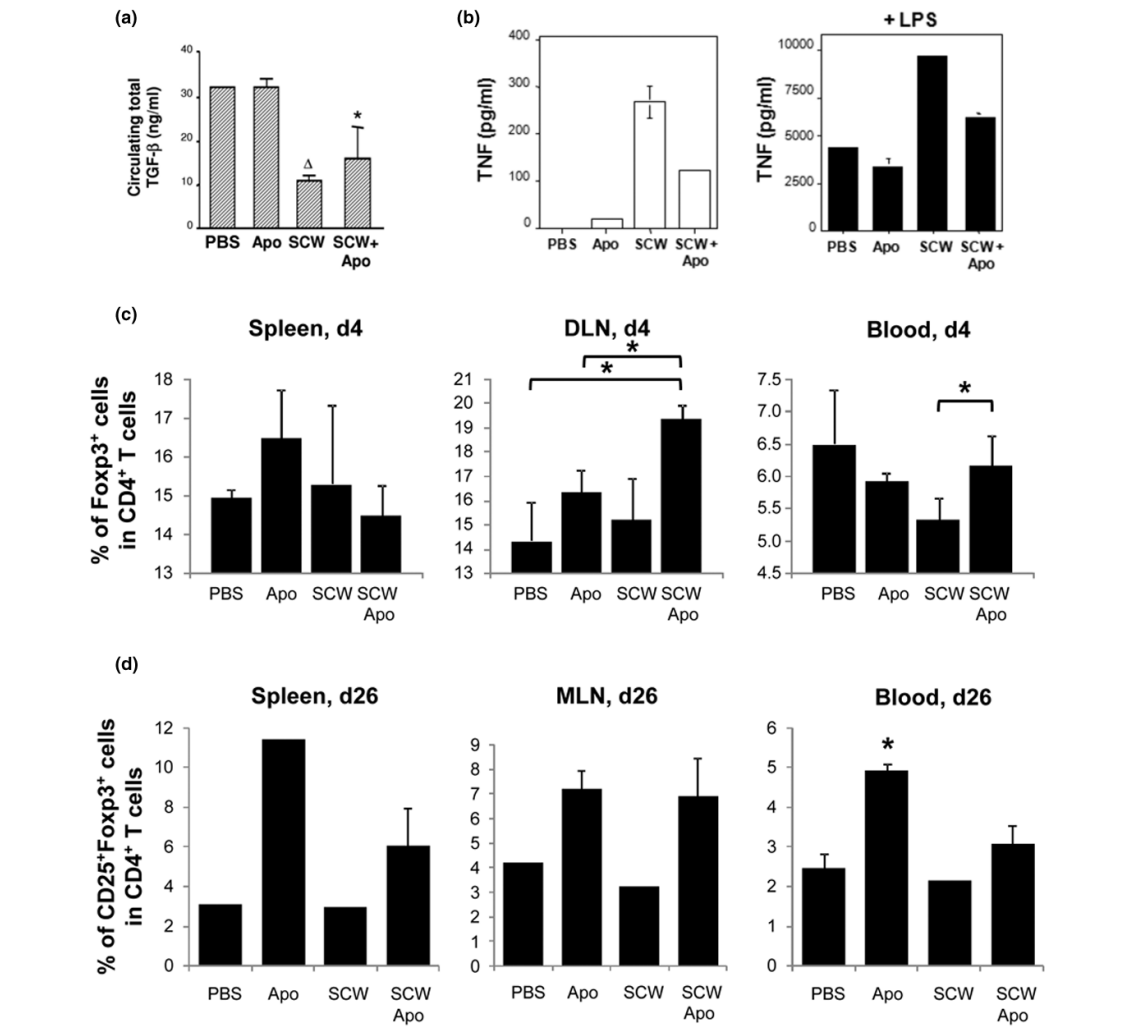
Apoptotic-cell injection prevents streptococcal cell wall-induced arthritis by macrophageactivation prevention and regulatory T cells increase. **(a) **Rats from the different groups were punctured into the retro-orbital sinus at day 1 to quantify circulating total transforming growth factor beta (TGFβ) by ELISA in the serum (mean ± standard error of the mean (SEM); n = 3 rats per group, excepted PBS n = 2). Apo, apoptotic cells; SCW, streptococcal cell wall. ^Δ^*>P *< 0.01 and *P < 0.05 compared with PBS-injected rats. **(b) **Macrophages issued from rats of the different groups were harvested from the peritoneum cavity 4 days after injection. TNF (mean ± SEM of the duplicate measurements) was tested by ELISA in the supernatant of the cultured macrophages (1 × 10^6 ^cell per condition) from rats from each group (n = 3 to 4 rats) untreated (left panel) or after lipopolysaccharide (LPS) (50 ng/ml) overnight stimulation (right panel). Experiment repeated twice with similar results. **(c) **At day 4 and **(d) **at day 26 after SCW immunization, rats were sacrificed and the blood, spleen, inguinal lymph nodes (DLN) and mesenteric lymph nodes (MLN) were collected to analyze Foxp3^+ ^regulatory T cells by flow cytometry. Results expressed as mean ± SEM; three animals/group; *P < 0.05. (c) Results for MLN and spleen expressed as mean ± SEM of the duplicate experiments, corresponding to two or three rats pooled together and repeated twice, not allowing statistical analysis. *P < 0.05 compared with PBS-treated or SCW-treated rats (four to six individual animals). (d) Experiment was repeated twice with similar results.

Since apoptotic cells induced *in situ *have been demonstrated to increase the level of circulating TGFβ [8] and because previous studies have indicated that the systemic levels, not the local levels (that is, joints), of TGFβ were positively associated with the amelioration of SCW arthritis [17], we measured circulating TGFβ in the recipient rats at days 1, 4, 6 and 11 after induction of arthritis in the different conditions. Although circulating levels of total TGFβ between days 4 and 11 were not significantly modified in the different conditions tested, an increase of total TGFβ was observed in rats receiving apoptotic cells alone on day 11 (data not shown). The levels of total TGFβ, however, were significantly lower 1 day after injection in the SCW-injected groups (SCW vs. PBS, *P *< 0.01; SCW + apoptotic cells vs. PBS, *P *< 0.05; Figure 2a). To better appreciate the effects on TGFβ, the active form of circulating TGFβ was then measured on days 4 and 11 after SCW injection. Although no statistical differences between the various groups were observed (day 4, mean ± standard error of the mean: PBS, 96.3 ± 9.4 pg/ml; apoptotic cells, 242.5 ± 96.2 pg/ml; SCW, 171.6 ± 103.5 pg/ml; apoptotic cells + SCW, 157.0 ± 30.7 pg/ml; two to six rats per group), an increase of active TGFβ was seen at day 4 in rats receiving apoptotic cells alone.

Since apoptotic cell injection reduced the severity of arthritis induced by SCW immunization and macrophages involved in apoptotic cell capture exhibited anti-inflammatory features [23], we investigated *ex vivo *macrophage functional characterization as assessed by the levels of the inflammatory cytokine TNF. Macrophages from the peritoneum cavity of rats receiving SCW alone, apoptotic cells alone or SCW plus apoptotic cells were enriched at day 4 and cultured overnight. TNF was tested in the culture supernatant. SCW immunization induced a marked activation of peritoneal macrophages as demonstrated by a strong spontaneous secretion of TNF compared with rats receiving only PBS or apoptotic cells (Figure 2b, left panel). Injection of apoptotic cells with SCW decreased spontaneous TNF release in the culture supernatants compared with SCW only (Figure 2b, left panel).

To confirm these data, we then challenged the enriched macrophages from the indicated rats to determine their response to LPS stimulation. As expected, macrophages from PBS-treated rats produced TNF in response to LPS, slightly more than those from rats injected with apoptotic cells alone 4 days earlier (Figure 2b, right panel). Macrophages from SCW-injected rats produced increased levels of TNF in response to LPS. Injection of apoptotic cells with SCW prevented macrophage from LPS-induced TNF secretion (Figure 2b, right panel).

Co-injection of apoptotic cells to SCW therefore reduced SCW-induced macrophage activation *in vivo*. This reduction of activation may be related to apoptotic cell uptake.

### Apoptotic cell injection leads to increase in CD4^+^CD25^+^Foxp3^+ ^regulatory T cells

Uptake of apoptotic bodies by macrophages has been shown to induce Treg generation [8,13], so we investigated the role of such a Treg population in the control of SCW-induced arthritis by apoptotic cell injection. We assessed the Treg population based on their constitutive expression of the transcriptional factor Foxp3 in the blood, spleen, mesenteric and inguinal lymph nodes 4 and 26 days after arthritis induction.

Apoptotic cell injection by itself induced an increase of the percentage of Tregs among the CD4^+ ^T cells in all tested organs (including the spleen and draining inguinal lymph nodes) at day 4, and at day 26 in the spleen, mesenteric lymph nodes and considerably in the blood (Figure 2d; apoptotic cells vs. PBS or SCW, *P *< 0.01; apoptotic cells vs. SCW + apoptotic cells, *P *< 0.05). This observation confirms previous results obtained in mice [8,12,13,24]. Whereas injection of SCW did not induce any increase in the Treg population in all organs tested on any day – the percentage of Tregs in SCW-treated rats was similar to the percentage of Tregs in PBS-treated rats (Figure 2c, d) – injection of apoptotic cells with SCW immunization induced a significant increase in Tregs at day 4 in blood (*P *< 0.05 vs. SCW alone; Figure 2c, right panel). The Treg increase after apoptotic cell injection in SCW-treated rats was also observed in the draining inguinal lymph nodes without reaching statistical significance (*P *= not significant vs. SCW alone; Figure 2c, middle panel).

At day 26 in all of the organs tested – in particular, in the site of immunization with SCW (that is, the mesenteric lymph nodes) – apoptotic cell injection induced a marked increase of Tregs compared with SCW alone (Figure 2d, middle panel). Indeed, the Treg increase observed in the mesenteric lymph nodes at day 26, and not in the draining inguinal lymph nodes (data not shown), of rats injected with SCW plus apoptotic cells was as high as that observed in rats receiving only apoptotic cell injection. The prevention of and decrease in inflammation, joint swelling and bone destruction due to apoptotic cell injection is therefore associated with reduced TNF secretion by macrophage and Treg increase, especially at the inflammatory site.

## Discussion

Apoptotic cell injection has been previously shown to induce a transient immunosuppressive environment, sufficient in animal models to reduce inflammation [6,13] or to favor tolerance toward allo-antigens [13,22] or self antigens [8]. RA is an autoimmune disease characterized by a lack of apoptosis leading to hyperplasia of the synovial lining. The macrophage is one of the principal cell types that contribute to the pathogenesis of RA, since macrophage depletion suppresses the chronic phase of SCW-induced arthritis [21]. This is why we decided to infuse apoptotic cells in a RA model: providing apoptotic cells to macrophages may change their proinflammatory behavior. In the present article, we showed that apoptotic cell injection prevents macrophages from SCW-induced TNF secretion. In addition, apoptotic cell infusion leads to an increased of Tregs in the draining lymph nodes. This was associated with a decrease in the symptoms and the severity of SCW-induced arthritis.

Both acute and chronic joint inflammations were significantly inhibited by apoptotic cell injection – including reduction of swelling and decreased tissue and bone destruction. The synovial inflammatory cell infiltration and TNF production were also markedly suppressed. Our data indicate that delivery of apoptotic cells *in vivo *even in the periphery may initiate anti-inflammatory mechanisms to antagonize the joint inflammatory response. Macrophages exposed by apoptotic cells seemed to be less efficient to induce and sustain SCW inflammation. Indeed, apoptotic cell injection acts in two different ways. First, apoptotic cells together with phagocytes that digest apoptotic cells in a very efficient manner induce an anti-inflammatory microenvironment. This first sequential event directly targets the acute phase induced by the SCW complexes and may prevent effector T-cell activation and migration to inflammatory sites such as joints and bones. The apoptotic cell injection-induced TGFβ increase also correlates with the Treg increase. Then, after the uptake of apoptotic cells, professional phagocytes such as macrophages become more resistant to inflammatory signals [23,25] and cytokines, as we observed here with the decrease of TNF secretion after LPS stimulation. This second sequential event targets phagocytes and may prevent occurrence of the chronic phase. This is in line with the work of Richards and colleagues showing that macrophage depletion alters the chronic phase of SCW-induced RA [21].

Macrophages, after the uptake of apoptotic cells, may then release TGFβ- which we detected in the periphery very early after apoptotic cell infusion – and may contribute to the resistance of macrophages to LPS stimulation, as previously described [23]. The reduction of circulating TGFβ in SCW-induced inflammation at day 1 and the slight increase in circulating active TGFβ at day 4 in apoptotic cell-treated animals at the time of articular index reduction also suggests a critical role for endogenous TGFβ in the control of inflammation. The Treg increase observed at day 4 in the inguinal draining lymph nodes of animals receiving apoptotic cells plus SCW also supports this point. The increase of TGFβ we observed in the circulation after apoptotic cell injection alone is in line with another experimental model, where apoptotic cells were induced *in vivo *and led to a TGFβ increase for 4 days with a peak at 24 hours after apoptotic cell induction [8]. As previously shown in tolerance induction by oral administration of SCW peptide [18], TGFβ is mainly responsible for the prevention of the disease; apoptotic cell administration may induce a similar effect.

In addition to macrophages, immature dendritic cells may also uptake apoptotic cells and then may produce less IL-1β, IL-6 and TNF in response to LPS stimulation [4] – all of these proinflammatory cytokines were found at elevated levels in RA patients or in collagen-induced arthritis mice. The effects may be ascribed at least in part to the TGFβ production by immature dendritic cells upon digestion of apoptotic cells [26]. Moreover, IL-1β has been demonstrated as an important mediator of SCW-induced arthritis by promoting Th17 differentiation [27]. One may speculate that apoptotic cell infusion by downregulating IL-1β production in responses to inflammatory signals [4] controls Th17 response and subsequent arthritis development.

The second effect mediated by apoptotic cell injection may implicate the release of TGFβ, as described previously [3,13,23]. The elevated concentration of TGFβ permits the Treg increase, preventing activation of specific T cells responsible for the chronic phase of arthritis. The fact that the Treg increase was observed in our model only in the lymph nodes draining SCW-induced pathology further supported this idea. In line with this notion, it has been shown that adoptive transfer of CD25^+ ^Tregs effectively decreases collagen-induced arthritis [28]. Because Th17 cells has been suggested to be involved in the induction of arthritis in an experimental model of spontaneous arthritis [29,30], apoptotic cell injection may also increase T-cell polarization to Tregs instead of Th17 differentiation by increasing the TGFβ levels.

## Conclusions

In the present article we have shown that apoptotic cell injection can significantly decrease the occurrence and the severity of SCW-induced RA. Apoptotic cell injection offers a tool to control and prevent macrophage-induced SCW inflammation. Apoptotic cell prevention of SCW-induced RA seems to be achieved sequentially: first after uptake of apoptotic cells by phagocytes, in particular macrophages that decrease their response to LPS; and then through a Treg increase in lymphoid organs, in particular in the draining lymph nodes, thus preventing and controlling SCW inflammation. These findings may provide insight into understanding the pathogenesis of chronic inflammation and autoimmune disease, and may also offer clues to manipulate Tregs and macrophages by apoptotic cell injection. The data are in line with our previous work suggesting the potential of apoptotic cells to treat ongoing autoimmune disease such as experimental autoimmune encephalomyelitis.

## Abbreviations

BSA: bovine serum albumin; DMEM: Dulbecco's modified Eagle's medium; ELISA: enzyme-linked immunosorbent assay; FBS: fetal bovine serum; Foxp3: forkhead box P3; H & E: hematoxylin and eosin; IL: interleukin; LPS: lipopolysaccharide; mAB: monoclonal antibody; PBS: phosphate-buffered saline; RA: rheumatoid arthritis; SCW: streptococcal cell wall; Treg: regulatory T cell; TGFβ: transforming growth factor beta; TNF: tumor necrosis factor.

## Competing interests

The authors declare that they have no competing interests.

## Authors' contributions

SP designed and performed most of the experiments and wrote the manuscript. PS participated in the writing of the manuscript. WJC initiated and directed the study, designed and performed some of the experiments and edited the manuscript. All authors read and approved the final manuscript.
